# New insights into the genetic loci related to egg weight and age at first egg traits in broiler breeder

**DOI:** 10.1016/j.psj.2024.103613

**Published:** 2024-03-05

**Authors:** Xiaochun Ma, Fan Ying, Zhengda Li, Lu Bai, Mengjie Wang, Dan Zhu, Dawei Liu, Jie Wen, Guiping Zhao, Ranran Liu

**Affiliations:** ⁎ State Key Laboratory of Animal Biotech Breeding; State Key Laboratory of Animal Nutrition and Feeding; Key Laboratory of Animal (Poultry) Genetics Breeding and Reproduction, Ministry of Agriculture; Institute of Animal Science, Chinese Academy of Agricultural Sciences, Beijing 100193, China; † Foshan Gaoming Xinguang Agricultural and Animal Industrials Corporation, Foshan 528515, China

**Keywords:** egg weight, age at first egg, genome-wide association analysis, meta analysis, broiler breeder

## Abstract

Egg weight (**EW**) and age at first egg (**AFE**) are economically important traits in breeder chicken production. The genetic basis of these traits, however, is far from understood, especially for broiler breeders. In this study, genetic parameter estimation, genome-wide association analysis, meta-analysis, and selective sweep analysis were carried out to identify genetic loci associated with EW and AFE in 6,842 broiler breeders. The study found that the heritability of EW ranged from 0.42 to 0.44, while the heritability of AFE was estimated at 0.33 in the maternal line. Meta-analysis and selective sweep analysis identified two colocalized regions on GGA4 that significantly influenced EW at 32 wk (**EW32W**) and at 43 wk (**EW43W**) with both paternal and maternal lines. The genes *AR, YIPF6*, and *STARD8* were located within the significant region (GGA4: 366.86-575.50 kb), potentially affecting EW through the regulation of follicle development, cell proliferation, and lipid transfer etc. The promising genes *LCORL* and *NCAPG* were positioned within the significant region (GGA4:75.35-75.42 Mb), potentially influencing EW through pleiotropic effects on growth and development. Additionally, 3 significant regions were associated with AFE on chromosomes GGA7, GGA19, and GGA27. All of these factors affected the AFE by influencing ovarian development. In our study, the genomic information from both paternal and maternal lines was used to identify genetic regions associated with EW and AFE. Two genomic regions and eight genes were identified as the most likely candidates affecting EW and AFE. These findings contribute to a better understanding of the genetic basis of egg production traits in broiler breeders and provide new insights into future technology development.

## INTRODUCTION

Chicken is an important source of high-quality protein for human production and life (Siegel, 2014). To meet this demand, fast-growing, white-feathered broilers were bred since 1950, and continuous genetic selection has been carried out to improve the production capacity (Muir et al., 2008; Rubin et al., 2010). However, compared to the extensively studied growth and feed efficiency traits, there is still relatively limited research on the reproductive traits of broiler breeders in genetic and breeding studies (Liu et al., 2022; Wen et al., 2022; He et al., 2023). Ding et al. (2022) conducted a comprehensive study on the egg production traits of broiler breeders at various stages. However, the emphasis on egg weight (**EW**) and age at first egg (**AFE**) traits in broiler breeders is still insufficient.

The EW, an important economic trait, is one of the most important indicators for assessing poultry reproductive performance. The EW is also a highly heritable quantitative trait (Savegnago et al., 2011), with estimates of the heritability ranging from 0.48 and 0.64 (Hartmann et al., 2003; Kamali et al., 2007). Furthermore, with increasing hen age, EW continuously increases throughout the laying period. However, both oversized and undersized eggs can cause a series of problems in the poultry industry, such as affecting the egg production rate, hatchability, chick fitness, and egg quality (Liu et al., 2018). Therefore, controlling EW is very important for improving poultry reproductive performance and economic benefits. A total of 393 quantitative trait loci (**QTLs**) associated with EW have been reported on 16 different chromosomes (15 autosomes and one sex chromosome) and included in the Animal QTL database (https://www.animalgenome.org/cgi-bin/QTLdb/index).

The AFE is one of the indicators directly related to egg traits. It is determined by genetic factors, and early studies have shown that the estimated heritability of AFE ranges from 0.20 to 0.56 in different strains (Hu et al., 2004; Lwelamira et al., 2009). The AFE, a complex quantitative trait regulated by multiple minor genes, involves the entire hypothalamic-pituitary-gonadal axis. Gonadotropin-releasing hormone (**GnRH**) initiates the cascade reaction by binding to its receptor, stimulating the synthesis and secretion of gonadotropins, inducing the synthesis of gonadal steroids, and ultimately leading to the start of follicular development, ovulation, and egg production in hens (Xu et al., 2011). To improve egg production, is important to control when chickens start laying to ensure they are at the appropriate age.

The GWAS analysis has many advantages, but thus far, many studies have only utilized phenotypes from individual lines at single time points (Gu et al., 2011; Wolc et al., 2012; Sun et al., 2013; Tan et al., 2023). In practical breeding research, some traits can be measured repeatedly. This advantage of repeated measurements provides an opportunity to identify time-related and consistent genetic loci (Smith et al., 2010; Zhang et al., 2013). Additionally, meta-analysis across different populations greatly improves the reliability and universality of the analysis results.

In this study, we conducted single-variant GWAS, meta-GWAS, and selective sweep analyses on 6,842 chickens from both paternal and maternal lines using an imputed 55K chicken SNP array with 10X resequencing data. We determined the genetic relationship between EW at different ages and AFE. The main goal of our current work was to precisely locate the associated genes that influence the phenotypic variation of AFE and EW and provide valuable insights into the genetic basis of these traits.

## MATERIALS AND METHODS

### Experimental Birds

A total of 6,842 broiler breeder were obtained from both paternal and maternal lines from Foshan Gaoming Xinguang Agricultural and Animal Industrials Co., Ltd. (Foshan, China). The paternal line chickens reached a body weight of 3.1 kg at 45 d of age, while the maternal line chickens reached a body weight of 2.5 kg at 42 d of age. Chickens of both lines achieved a feed conversion ratio below 1.70 from 28 to 42 d of age.

Female lines have been selected for egg-laying traits for 10 generations. In our analyses 4,784 genotyped individuals from the 8th, 9th and 10th generations were used. The paternal line, on the other hand, has not undergone any selection for egg production traits, and their selection was primarily toward growth and feed efficiency traits. All experimental individuals were transferred to individual cages in the laying house when they were approximately 140-days-old and were fed, vaccinated, and managed following standard procedures for broiler breeders. The chickens used in the experimental analysis had complete genomic information. Blood samples were collected from the brachial veins of each chicken using the breeding program's standard procedure, which was approved by the Animal Welfare and Ethics Committee of the Institute of Animal Sciences, Chinese Academy of Agricultural Sciences (IAS-CAAS, Beijing, China).

### Phenotypic Measurements

To accurately describe the genetic basis of EW during the laying period, we measured EW at two time points, 32 and 43 wk. The average EW value of each individual was calculated for subsequent experiments. We recorded the number of eggs laid at 43 and 49 wk. We used a barcode scanner and an egg-laying recording system to record the number of eggs laid by each chicken at a fixed time in the morning of each day. We calculated the age at laying based on the time of the first egg recorded for each chicken. Individuals that deviated from the mean by more than 3 standard deviations were excluded for subsequent analyses.

### Genotyping, Imputation, and Quality Control

A total of 6,842 individuals from the paternal and maternal lines were genotyped using the 55 K SNP genotyping arrays developed by the Institute of Animal Sciences, Chinese Academy of Agricultural Sciences (completed by Beijing Compass Biotechnology Co., Ltd.) (Liu et al., 2019a). First, PLINK v1.9 (Chang et al., 2015) software was used to perform quality control on 54,130 SNPs from the 6,842 individuals. SNPs with a minor allele frequency (**MAF**) of ≥ 5% and SNP call rate of ≥ 90% were retained, as well as those with an individual call rate of ≥ 90%. Ultimately, 42,262 SNP loci on chromosomes 1-28 and Z were retained for subsequent analyses. The missing loci were genotyped and imputed in using Beagle 5.2 software (Browning et al., 2018) after the genotypic variants were eliminated.

A total of 150 individuals randomly selected from the two lines were subjected to whole-genome sequencing (**WGS**) using the Illumina NovaSeq 6,000 platform. The sequencing depth of each library was not less than 10X using the latest GRCg7b reference genome. PLINK v1.9 software was used for quality control, with criteria of individual call rate ≥ 90%, SNP call rate ≥ 90%, and MAF ≥1%. After screening, a total of 11,220,611 SNP markers from the 150 sequenced individuals were retained for subsequent analysis.

We imputed the chicken 55K genotyping data to the level of whole-genome sequencing using Beagle 5.2 software. A reference panel of 150 whole-genome sequenced individuals and a target panel of 6,482 55K SNP genotyping individuals were used for this analysis. Since the reference panel cannot have missing genotypes, we first used the default parameters of Beagle 5.2 software to impute the resequencing data (Li et al., 2020). Then, the 55K SNP chip data were imputed to the resequencing level using default parameters. The accuracy of genotype imputation was evaluated using the squared correlation coefficient (R^2^) of the imputed genotypes with the true genotypes, which was provided by Beagle 5.2. To ensure the accuracy of imputation, strict quality control was conducted for each SNP based on the research results of (Sahana et al., 2016), using BCFtools-1.16 software to retain SNPs with imputed allele R^2^ ≥ 0.9 and MAF ≥ 0.05. We calculated the allele R^2^ at the chromosome level for the 2 lines after quality control, with an average R^2^ value of ≥ 0.96. Finally, a total of 7,795,273 SNPs and 6,842 individuals were used for the subsequent GWAS.

### Population Structure Testing

First, we conducted principal component analysis (**PCA**) using PLINK v1.9 software to analyze the paternal and maternal lines. The first two principal components explained a cumulative total variance of 25%. Additionally, we analyzed the population structure using admixture -1.3.0 (Chimusa et al., 2020). To better evaluate the genetic characteristics between the two populations, we quantified and plotted the LD decay level using PopldDecay software (Zhang et al., 2019), with a maximum distance of 500 kb.

### Estimation of Heritability and Genetic Correlations

Genetic parameters were estimated using the restricted maximum likelihood (**REML**) animal model for traits and two traits, and the statistical model equation was as follows:

(1) y=Xb+Zα+e

where y is a vector of phenotypic values for egg-laying traits; X is the design matrix that associates observations with the appropriate combination of fixed effects, Z is the design matrix that associates observations with the appropriate combination of random effects, with generation and batch added to the model as fixed effects in this study, b is a vector of fixed effects α is a vector of random effects, and e is the vector of residual errors. The variance‒covariance matrix for the random effects is as follows:

(2) $$var\left[\begin{array}{c} a\\ e \end{array}\right]=\left\lfloor \begin{array}{cc} G\sigma_{a}^{2} & 0\\ 0 & I\sigma_{e}^{2} \end{array}\right\rfloor \;$$

where$\sigma_{a}^{2}$ and $\sigma_{e}^{2}$ represent the additive genetic variance and the residual environmental variance, respectively; G is the genomic relationship matrix constructed using SNP markers; and I is the unit matrix. Heritability is calculated as $\sigma_{a}^{2}/\left(\sigma_{a}^{2}+\sigma_{e}^{2}\right)$.

Genetic correlation and phenotypic correlation analyses for this trial were calculated by a two-trait model, which was as follows:

(3) $$\left[\begin{array}{c} y_{1}\\ y_{2} \end{array}\right]=\left[\begin{array}{cc} X_{1} & 0\\ 0 & X_{2} \end{array}\right]\left[\begin{array}{c} b_{1}\\ b_{2} \end{array}\right]+\left[\begin{array}{cc} Z_{1} & 0\\ 0 & Z_{2} \end{array}\right]\left[\begin{array}{c} a_{1}\\ a_{2} \end{array}\right]+\left[\begin{array}{c} e_{1}\\ e_{2} \end{array}\right]$$

where y,X,b,Z,α and e are the same formula (2).

The GBLUP method was used for genetic parameter estimation, the G-matrix was constructed by VanRaden's method (VanRaden, 2008), The calculation process and model fitting were completed using ASReml v4.1 in the R v4.1.2 software. (Gilmour et al., 2015).

### Genome Wide Association Study

Generalized paired analyses were performed for the three traits on 6,842 individuals from the paternal and maternal lines using univariate linear mixed models with GCTA_1.93 software (Yang et al., 2011). The statistical model used was as follows:

(4) $$y=W_{\alpha }+x\beta +u+\varepsilon ;u∼MVN_{n}\left(0,\lambda \tau^{-1}K\right),\in ∼MVN_{n}\left(0,\tau^{-1}I_{n}\right)$$

where y represents the phenotype vector; W is the indicator matrix of the fixed effects, with the first column being 1 and subsequent columns being the values of fixed effects or covariates, and α is the coefficient vector of the fixed effects. x represents the genotype vector, β is the SNP effect, u is the random effect vector, and ϵ is the residual vector. $\text{MV}N_{n}$ denotes the n-dimensional multivariate normal distribution, λ is the ratio of 2 variance components, $\tau^{-1}$ is the residual variance, K is the kinship matrix based on SNPs, and $I_{n}$ is the unit matrix.

Considering the extensive linkage disequilibrium (**LD**) among SNPs, Bonferroni correction was excessively conservative. Therefore, PLINK software was utilized with parameters –indep-pairwise 25 5 0.2 to perform independent tests on the SNPs, with an R^2^ threshold of 0.2 (Liu et al., 2019b). The threshold of genome-wide significant *P* values was adjusted based on the number of independent SNPs. Manhattan and Q‒Q plots were created for each trait with the CMplot package in R (version 4.1.2).

### Meta Analyses

Compared to performing GWAS analysis directly on 2 line-specific datasets, meta-analysis incurs minimal efficiency loss and helps mitigate the impact of population stratification on GWAS results (Willer et al., 2010). Meta-analysis is widely employed to combine summary data of many genetic variations across the genome in GWASs. In this study, Metal software (version 2011-03-25) was used to perform meta-analysis on three traits of the paternal and maternal lines.

### Identification of Selective Sweeps

In this study, a combination of 2 methods, the population differentiation index (*F*_ST_) and nucleotide polymorphism (π) methods, was employed to uncover genomic regions under selection. The software VCFTools v0.1.13 was used to calculate the π and *F*_ST_ results for the paternal and maternal populations (Danecek et al., 2011), with a window size of 10 kb. The results were visualized using the ggplot2 package in R (version 3.4.4).

### Linkage Disequilibrium and Haplotype Analysis

The candidate region was extracted using PLINK v1.9, and a custom script was employed to format the extracted SNP data accordingly. LDBlockShow v1.39 software was used to analyze the level of LD within the candidate region (Dong et al., 2021). LD blocks were defined following the criteria established by Barrett et al. (2005). The GLM was employed to analyze the impact of each haplotype on the EW and AFE, with a significance threshold set at *P* < 0.05. The variant effect predictor (VEP) (McLaren et al., 2010) provided by Ensembl (http://www.ensembl.org) was used to determine the annotations of candidate genes adjacent to significant SNPs.

## RESULTS

### Basic Statistics and Genetic Parameters

The basic statistics of trait distribution for 6,842 broiler breeders are presented in (Table 1). The EW32W and EW43W of the maternal and paternal lines were located within the range of 58.2 to 61.5 g and 66.6 to 67.9 g, respectively. The mean AFE was 183.5 d in the maternal line and 193.0 d in the paternal line, with significant phenotypic differences observed between the 2 lines.

**Table 1:** Descriptive statistics of phenotypes in the paternal and maternal lines of broiler breeders.

Lines^1^	Traits^2^	n^3^	Max	Min	Mean	SD^4^	CV (%)^5^
Paternal	AFE	1,813	221	172	193	8.2	4.3
	EW32W	2,058	76.0	45.2	61.5	4.7	7.6
	EW43W	1,762	83.8	50.7	67.9	5.0	7.4
Maternal	AFE	4,067	215.0	163.0	183.5	8.2	4.5
	EW32W	4,784	79.1	41.0	58.2	4.4	7.5
	EW43W	3,728	80.8	50.7	66.6	4.4	6.6

1 Paternal and maternal represent 2 broiler breeder lines.

2 AFE stands for age at first egg; EW32W and EW43W stand for egg weight at 32 wk of age and 43 wk of age.

3 Number of samples involved in the analysis.

4 SD stands for standard deviation.

5 CV stands for coefficient of variation.

Genetic parameters, including heritability and correlation coefficients, for EW32W, EW43W, AFE, egg production at 43 wk, and egg production at 49 wk were estimated using a kinship matrix constructed from 4,784 maternal line samples genotyped with the 55K SNP chip. The AFE and egg production exhibited low to moderate heritability (0.16-0.33), while EW displayed moderate to high heritability (0.42-0.44). There was a weak positive correlation (0.08-0.16) between AFE and EW. However, there was a strong positive correlation (0.93) between EW32W and EW43W. Additionally, there was a strong positive correlation (0.98) between egg production at 43 and 49 wk. Conversely, there was a moderate to strong negative correlation (0.25-0.69) between egg production and AFE as well as EW (Table 2).

**Table 2:** Estimates of genetic parameters for egg production traits in 4,784 hens of the maternal line of broiler breeders.

Traits	AFE	EW32W	EW43W	EN43W	EN49W
AFE	**0.33 (0.02)**	0.06 (0.02)	0.07 (0.02)	−0.40 (0.08)	−0.32 (0.02)
EW32W	0.16 (0.05)	**0.42 (0.02)**	0.62 (0.01)	−0.09 (0.02)	−0.07 (0.02)
EW43W	0.08 (0.06)	0.93 (0.02)	**0.44 (0.02)**	−0.16 (0.02)	−0.16 (0.02)
EN43W	−0.69 (0.05)	−0.25 (0.07)	−0.27 (0.07)	**0.20 (0.02)**	0.93 (0.003)
EN49W	−0.56 (0.06)	−0.25 (0.07)	−0.28 (0.08)	0.98 (0.01)	**0.16 (0.02)**

Heritability on the diagonal, genetic correlations above the diagonal and phenotypic correlations down the diagonal, SE in parentheses.

GWAS analysis was conducted on all traits using imputed genotype data, and independent testing was performed on all SNPs across all chromosomes. Genome-wide suggestive thresholds and genome-wide significance thresholds for each trait were *P* < 1.00/Indep SNP and P < 0.05/Indep SNP (Table 3), respectively.

**Table 3:** Basic statistics of GWAS analysis data for paternal and maternal lines of broiler breeders.

Lines^1^	Traits^2^	n^3^	SNP N^4^	Indep SNP^5^	Significance^6^	Suggestive^7^
Paternal	AFE	1,814	8,267,081	487,402	1.03E-07	2.05E-06
	EW32W	2,058	8,267,081	486,190	1.03E-07	2.06E-06
	EW43W	1,762	8,267,081	486,866	1.03E-07	2.05E-06
Maternal	AFE	4,078	7,795,273	497,635	1.00E-07	2.01E-06
	EW32W	4,784	7,795,273	496,891	1.01E-07	2.01E-06
	EW43W	3,728	7,795,273	498,148	1.00E-07	2.01E-06

1 Paternal and maternal represent 2 broiler breeder lines.

2 AFE stands for age at laying, 32 W and 43 W for weekly age and EW for egg weight.

3 Number of individuals with the phenotype.

4 Number of SNPs used in this study.

5 Number of SNPs remaining after effective independent tests.

6 *P* value threshold for genome-wide significance.

7 *P* value threshold for genome-wide suggestive significance.

### Imputation Accuracy

**Figure 1:**
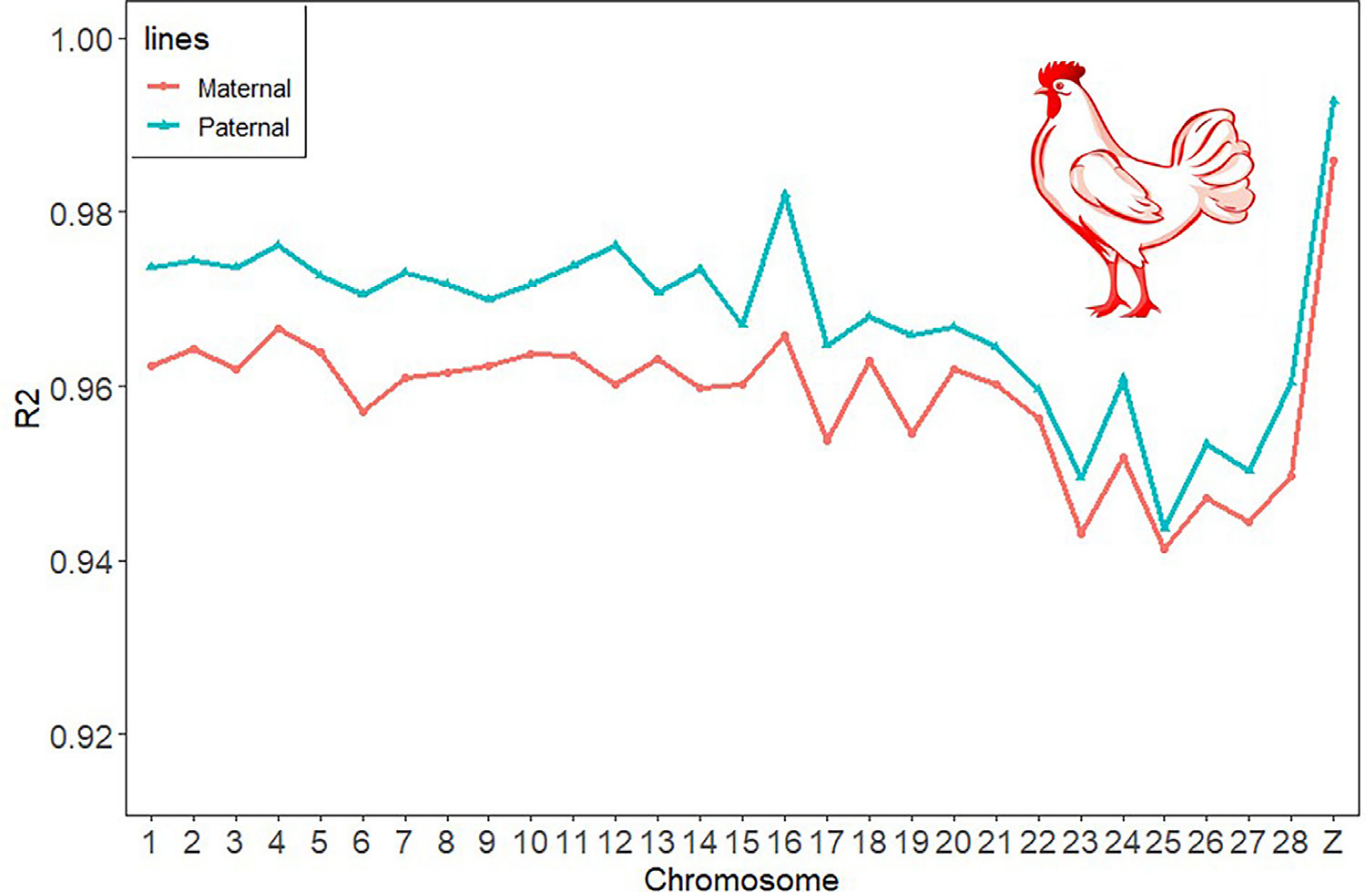
Accuracy of populated WGS data by chromosome for 6,842 broiler breeders.

To comprehensively evaluate the genotype imputation accuracy for the 6,842 individuals in the paternal and maternal lines, we summarized the average R^2^ values of the chromosomes of both lines (Figure 1). At the overall population level, the correlation between imputed genotypes and true genotypes was higher in the paternal line than in the maternal line. At the chromosome level, the R^2^ values decreased slightly but remained within a range of 0.96 to 0.98. Additionally, based on the imputed SNP density plot (Figure S1), all chromosomes were effectively imputed.

### Population Structure Testing

**Figure 2:**
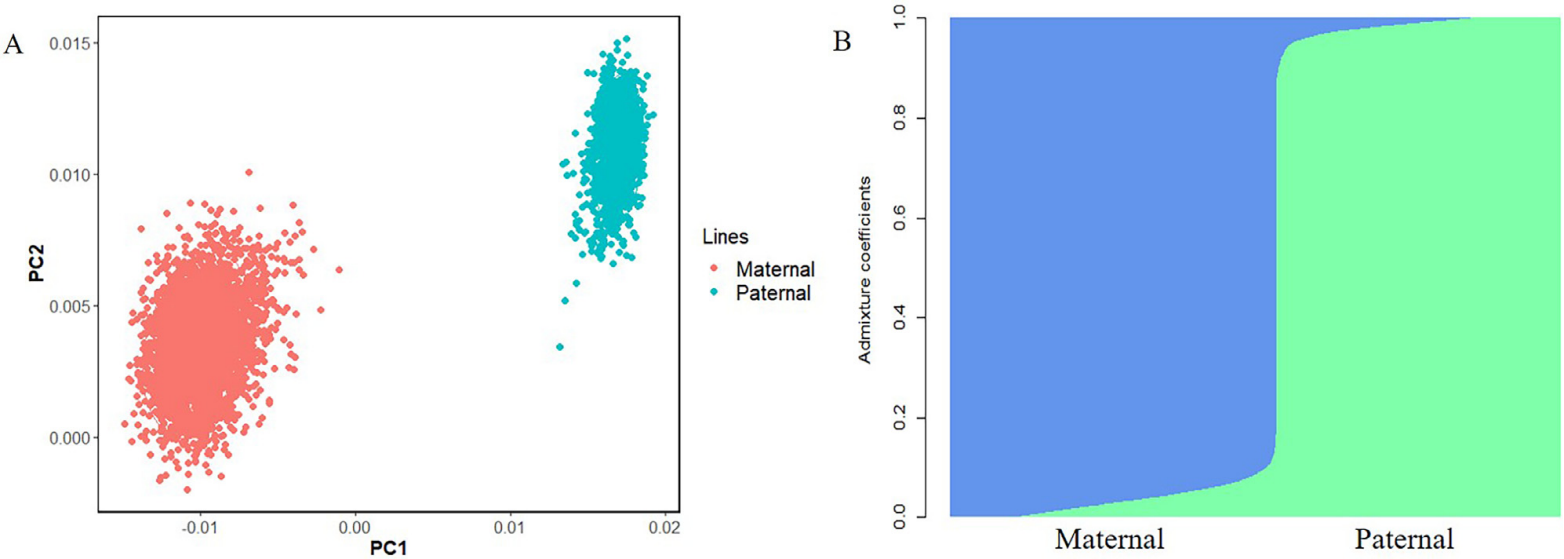
(A) Population structure analysis (PCA): the PCA plot depicting paternal and maternal lines of broiler chickens. Different colors represent different lines. (B) The PCA of the paternal and maternal lines, with the number of ancestral populations set to K=2.

A comprehensive analysis of the genetic relationship between the paternal and maternal lines was conducted. First, a PCA was performed on the paternal and maternal lineages using the 55K SNP chip (Figure 2A). Significant genetic differences were found between the two lines. Therefore, we treated the first three PCs as covariates and added them to the GWAS model as fixed effects. We further performed a genetic homogeneity analysis, and when assuming the number of shared ancestors (K) was 2, The integrated results are shown in Fig. S2. The population structure was consistent with the PCA results (Figure 2B). Moreover, the higher LD decay distance for the maternal lineage compared to the paternal lineage (Figure S3) indicates that the paternal lineage experiences greater selective pressure than the maternal lineage.

### GWAS for Single Line EW and AFE Traits

Individual GWAS analyses were performed for the three traits in the paternal and maternal lines separately. The SNPs were annotated using the Ensembl database. We identified 6 genes near significant SNPs that were potentially associated with AFE and 28 genes located on chromosome 11 that were potentially associated with EW (Table S1). To evaluate the reliability and accuracy of the GWAS results for both populations, we calculated the genomic inflation factor (λ), which ranged from 0.955 to 0.993, indicating adequate control of population structure (Figure S4).

### Meta‑Analysis for EW and AFE Traits

By performing single-trait GWAS analysis, we calculated the SNP effect sizes for the relevant traits. To further delineate the associated regions, we conducted a meta-analysis using all individuals from both the paternal and maternal lines.

**Figure 3:**
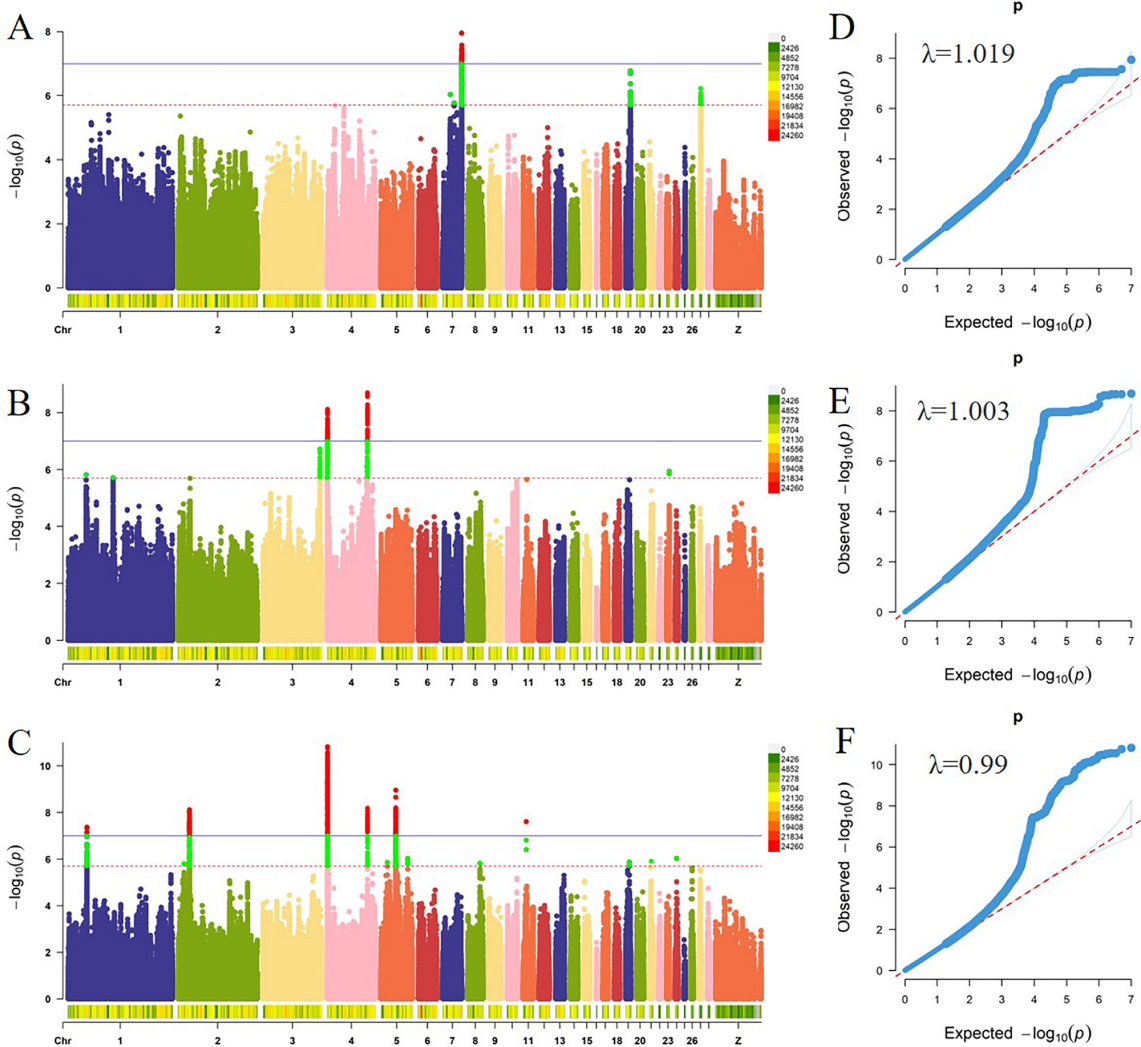
Manhattan and quantile–quantile plots of the meta-analyses for AFE and EW of the paternal and maternal lines of broiler chickens. Each dot represents one SNP. Plots A, B, and C in the Manhattan plots represent the results of the meta-analysis for AFE, EW32W, and EW43W in both the paternal and maternal lines. The QQ plots D, E, and F correspond to the respective Manhattan plots.

Meta-analysis was performed on EW for 2 chicken lines, which revealed 12 significant genomic regions on 11 chromosomes with suggestive signals for EW32W and EW43W (Table S2). Additionally, as depicted in Manhattan and QQ plots (Figures 3B and 3C), high genetic correlation indicated that these significant SNPs were primarily concentrated in two regions on GGA4. The genomic inflation factors (λ) ranged from 0.990 to 1.003, indicating effective control of population stratification.

For the EW32W trait, three significant intervals were identified on chromosomes 3 and 4. Chromosome 3 contained a 7.85 kb interval, where all significant SNPs were annotated to the *MTMR9* gene. Two intervals, sizes 252.53 kb and 1.24 Mb, were located on chromosome 4, with a total of 16 genes annotated to all significant SNPs, and the most significant SNP (chr4:75389203) was located in an intronic region of the *LCORL* gene (Table S2).

Regarding the EW43W trait, five significant intervals were identified on chromosomes 1, 2, 4, and 5. Two intervals, sizes 759.14 kb and 81.44 kb, were located on chromosome 4, with a total of 29 genes annotated to all significant SNPs, and the most significant SNP (chr4: 522234) was located in an intronic region of the *STARD8* gene. All significant SNPs were annotated (Table S2).

**Figure 4:**
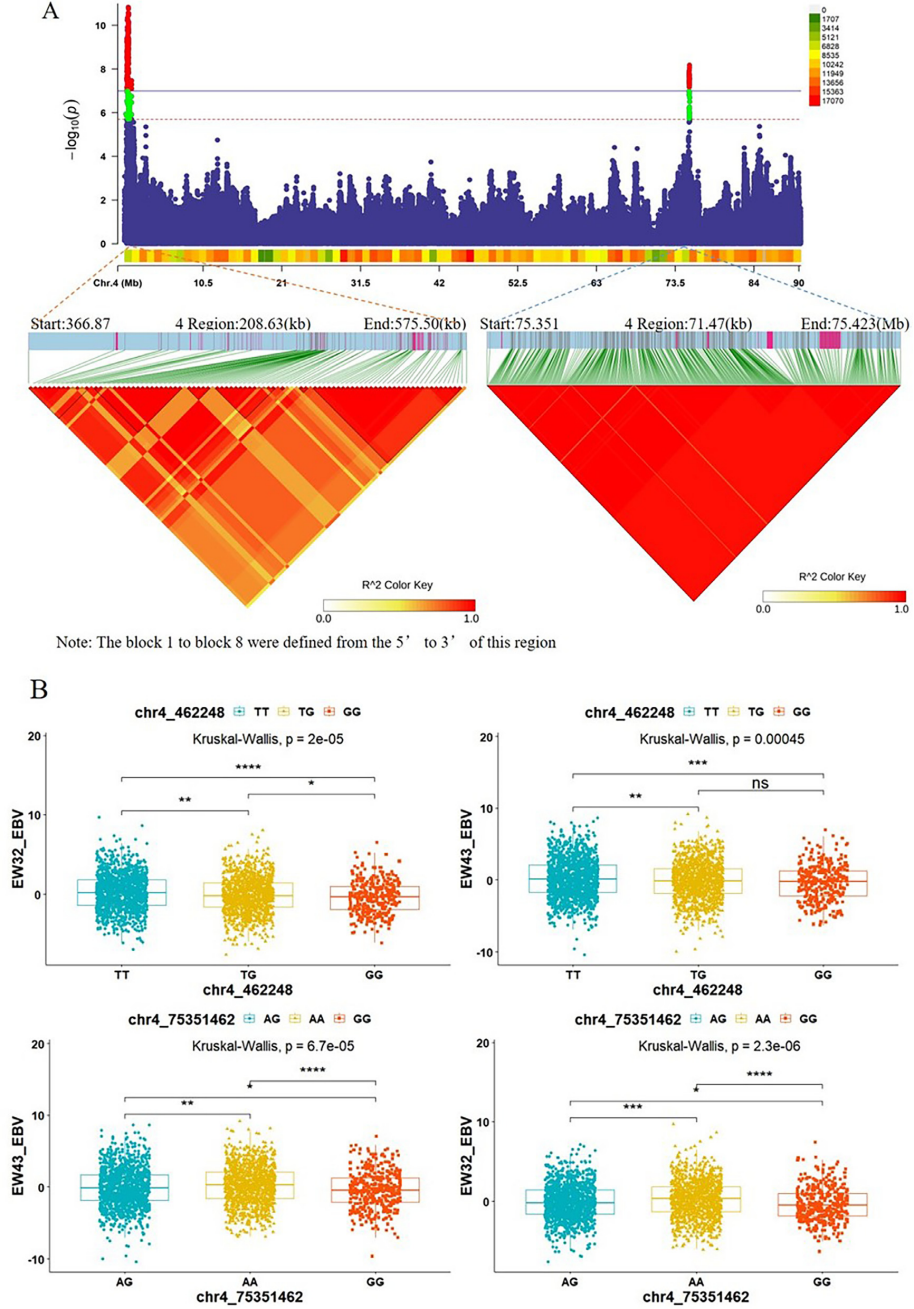
(A) LDBlock analysis of SNPs in the significant intervals (GGA4:366.87-575.50 Kb) and (GGA4:75.35-75.42 Mb) co-located after Meta-analysis of 32W and 43W egg weights, using 2,720 maternal line breeders. (B) Box plots were constructed with two Tag SNPs, 1 and 8, selected from eight blocks. Kruskal‒Wallis one-way ANOVA was used to test the effect of SNPs on EBV in EW32W and EW43W.

Considering the high heritability correlation (*r* = 0.93) between EW32W and EW43W, we conducted a comprehensive analysis of the Meta-GWAS results for both traits. We screened 2 colocated regions on chromosome 4, namely, GGA4: 366.86-575.50 Kb and GGA4: 75.35-75.42 Mb. Region 1 harbored genes such as *EDA2R, AR, OPHN1, YIPF6, STARD8*, and *RXFP2*, while region 2 contained genes such as *SLIT2, LCORL*, and *NCAPG*. We performed LDBlock analyses of these two regions using 2,720 individuals from the most recent generation of the maternal lines and found that region 1 contained 7 blocks with significant effects on EW breeding values. Region 2 was a highly linked area with significant LD that has a pronounced effect on EW EBV (Figures 4A and B and Table S3).

Genome-wide significant SNPs associated with AFE were successfully identified on chromosomes 7, 19, and 27 (GGA7, GGA19, and GGA27) (Table S2). These associated signals were primarily localized to 2 chromosomal regions, GGA7: 35.63-36.25 Mb and GGA19: 9.41-9.77 Mb, with multiple genes identified in each region. Significant SNPs on chromosome 27 were concentrated within a smaller interval and annotated to only two genes, *IGF2BP1* and *GIP*. Additionally, the overall meta-analysis yielded a low genomic inflation factor (λ=1.019), (Figure 3D). These results indicate that the observed genome-wide association signals, attributed to population stratification, can be disregarded.

**Figure 5:**
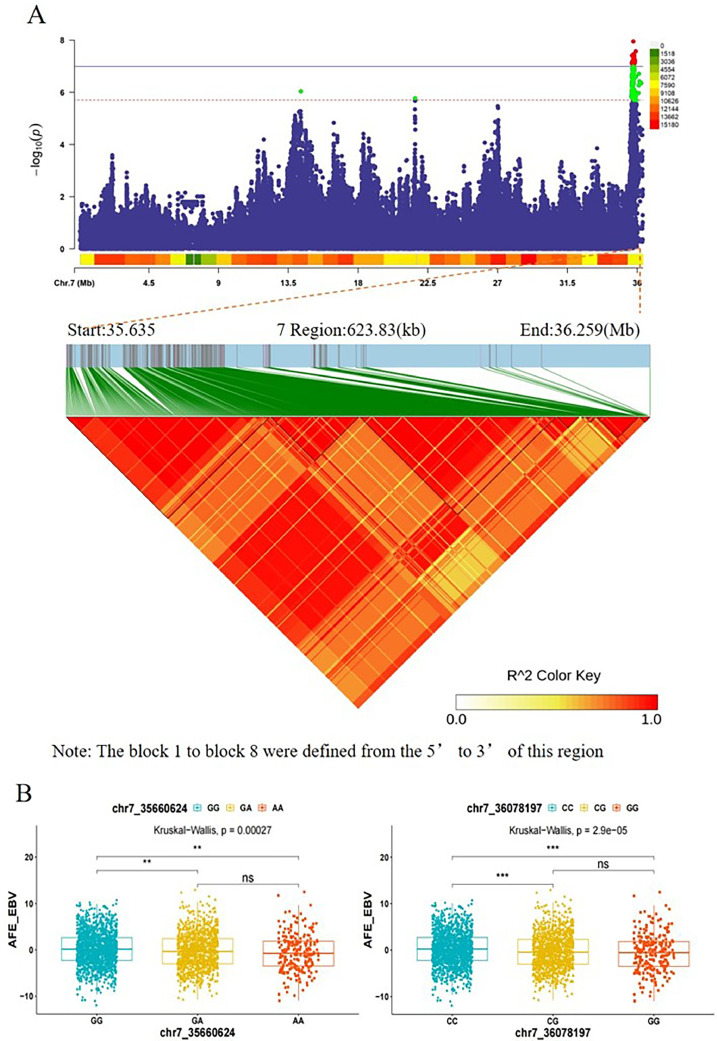
(A) LDBlock analysis of SNPs in significant intervals (GGA7:35.63-36.26 Mb) after meta-analysis of AFE using 2,720 chickens of the maternal line. (B) Box plots were constructed using Tag SNPs from two blocks, 1 and 8, selected from eight blocks. Effect of SNPs on EBV on day of whelping was tested by Kruskal‒Wallis one-way ANOVA.

Upon performing a meta-analysis on the results of the individual line GWAS, we annotated a total of 17 genes within the significant intervals. The most significant SNP (chr7:35905782) was located in an intronic region of the *ACVR1* gene. Considering the potential strong LD between neighboring variants, we conducted an LD block analysis on the most significant region using 2,720 individuals from the most recent generations of the maternal line. As a result, eight LD blocks were identified to be significantly associated with AFE breeding values, as depicted in (Figures 5A and 5B and Table S4).

### Selective Sweep Analysis

Considering the divergent breeding directions between the paternal and maternal lines, significant genomic differentiation and evident selection imprints were found between the 2 lineages. Exploring the differentiated genomic regions between the paternal and maternal lines was advantageous for the fine mapping of candidate intervals. We conducted an analysis of the regions under selection between the paternal and maternal lines using the *F*_ST_ and π ratio as indicators, aiming to precisely locate the candidate intervals.

For EW, we utilized *F*_ST_ > 0.2 as the criterion and simultaneously calculated the π ratio (π_p_/π_m_) for both paternal and maternal lines. A log_2_ (π ratio) < -1 or π ratio < 0.5 was considered the threshold. We analyzed the EW interval on chromosome 4, and within the interval (GGA4: 75.35-75.45 Mb), we identified a candidate region (75.44-75.45 Mb). Although the *F*_ST_ value did not reach statistical significance of 0.2, there was a noticeable trend toward selection in comparison to other regions. Moreover, the π ratio reached a significant level. To avoid potential loss of candidate regions, we considered this region as a candidate for further investigation, identifying the *LCORL* and *NCAPG* genes within it (Figure S5).

**Figure 6:**
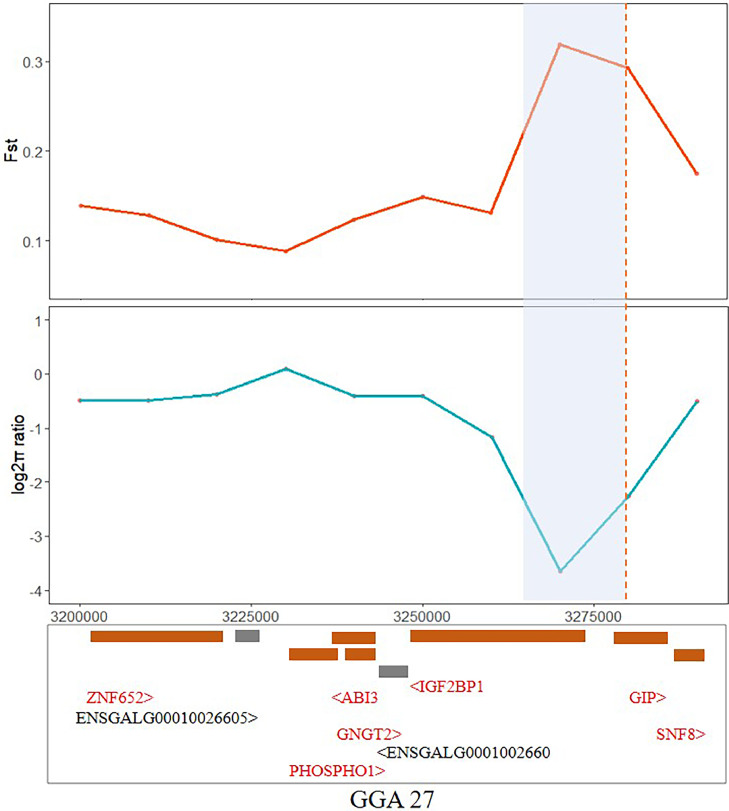
Results of selection signal analysis of AFE in the candidate interval of chromosome 27. Results of *F*_ST_ and π ratio analyses of paternal and maternal line.

Using the same criteria, we performed analysis on AFE and identified a candidate region on chromosome 27 (GGA27: 3.24-3.29 Mb). Within the 3.26-3.28 Mb region of the candidate interval, *F*_ST_ reached a significant level of differentiation. Calculation of the π ratio (π_p_/π_m_) revealed evident selection within this region in both lineages. We pinpointed the *IGF2BP1* gene in this region (Figure 6). Employing a similar approach, we screened a selected region (9.44-9.50 Mb) within the candidate interval on chromosome 19 (GGA19: 9.41-9.77 Mb) and identified the *MSI2* and *NF1* genes (Figure S6). Unfortunately, no candidate region was found on chromosome 7.

## DISCUSSION

EW and AFE are reproductive traits that can be measured early in the broiler breeder production process. Understanding their genetic basis is very important for selecting for breeding performance in meat-type chicken production. We estimated the genetic parameters for 4,784 hens in the maternal line. The heritability of EW32W and EW43W was high moderate (0.42-0.44), which is consistent with the results reported by Yi et al. (2015) who found heritabilities of EW at different stages ranging from 0.47 to 0.60. However, our results were slightly lower than those reported by Yi et al. (2015). Various factors contribute to the differences in heritability, including environmental influences, population size, animal breeds, differences in evaluation methods, and reliability recorded information.

The heritability of AFE was moderate, with a value of 0.33. Previous studies have shown significant variation in the heritability of AFE among different breeds, ranging from 0.20 to 0.56 (Lwelamira et al., 2009), which is consistent with our findings. Further analysis of the genetic correlation between traits revealed a strong positive genetic correlation between EW32W and EW43W (0.93), a low positive genetic correlation between AFE and EW32W (0.16), and a moderate to high negative genetic correlation between AFE and EW43W (0.25-0.69). These heritability estimates and genetic correlations are consistent with the results reported by Liu et al. (2018).

Meta-analysis is an increasingly important tool in GWASs of complex genetic traits (Willer et al., 2010). Ding et al. (2022) utilized GWAS results from 11,279 chickens and employed meta-analysis to localize a significant region on the Z chromosome associated with egg production.

In our study, we employed GWASs, meta-analyses, and selective sweep analyses to identify the SNPs and QTLs associated with EW and AFE traits in broiler breeders. Using these methods, we conducted GWAS analysis on the individual EWs at 2 time points and identified 2 genomic regions on GGA4, with sizes of 208.65 kb and 71.47 Kb, significantly associated with both EW32W and EW43W. LDBlock analysis of the significant SNPs in the 2 regions revealed a total of 8 LD blocks, containing seven genes associated with egg weight at both stages. Within interval 1 on GGA4, we located a gene, *AR*, related to chicken ovarian development. Huang et al. (2021) demonstrated in in vivo experiments with chickens that inhibiting *AR* significantly reduced the number of selected stage follicles and downregulated the expression of related genes in the follicles. This suggests that *AR* can regulate follicle selection by interacting with specific genes involved in chicken follicular development, highlighting its important role in follicle selection. The *YIPF6* gene can increase cell proliferation in conjunction with the *AR* gene (Djusberg et al., 2017). The *STARD8* gene has a lipid transfer function and is able to bind and transport cholesterol, phospholipids, and other lipid molecules, which are crucial for intracellular lipid metabolism and the regulation of lipid balance (Clark, 2012). It is also involved in cholesterol transport and metabolism, transferring cholesterol from the cell membrane to the endoplasmic reticulum to maintain normal cellular cholesterol levels. Lipids and cholesterol are important components of eggs (Puglisi and Fernandez, 2022). The regulation of lipid and cholesterol synthesis and deposition in chickens may lead to increased lipid content in the yolk, which in turn affects EW.

*NCAPG* and *LCORL* have been found to be associated with EW and body weight in many studies on poultry (Gu et al., 2011; Wolc et al., 2012). Niknafs et al. (2012) estimated the genetic correlation between the initial weight, 28-wk weight, 32-wk weight, and initial EW, 28-wk EW, and 32-wk EW of Mazandaran native chickens. They found that all EW traits were moderately positively genetically correlated (0.30-0.59) with weight-related traits. This suggests that there is likely a genetic basis for this relationship, and factors influencing one trait may also have an effect on the other. Considering the interaction between EW and body weight, larger chickens tend to produce larger eggs, and larger eggs can hatch chicks with higher body weights. We hypothesize that *NCAPG* and *LCORL* may have pleiotropic effects in both phenotypes. Our findings in this interval are consistent with the results of Yi et al. (2015). Additionally, the *NUP107* gene has been confirmed to play an important role in ovarian development and oocyte formation (França and Mendonca, 2022). During oocyte development, cell division and maturation occur, and nutrients accumulate within the oocyte, which can affect EW. Therefore, the quality of ovarian development directly impacts EW.

In addition, we identified 17 genes associated with AFE on three chromosomes, namely, chromosomes 7, 19, and 27. Functional annotation revealed that these genes are primarily involved in regulating neural system development, controlling early embryo development, and ovarian development, among other functions.

The most notable SNP was localized to the intronic region of the *ACVR1* gene. Studies in humans have demonstrated the pivotal role of *ACVR1* in ovarian function and folliculogenesis, as alterations in *ACVR1* can impact processes such as follicle selection, growth, and accumulation (Kevenaar et al., 2009). In a study conducted on mice lacking the *ACVR1* gene, there was a delay in embryo invasion and implantation, and uterine stromal cells failed to undergo decidualization, resulting in infertility (Komatsu et al., 2007). Therefore, it is hypothesized that the *ACVR1* gene may exert a similar role in the growth and development of chickens, and any disruptions in its expression could lead to delayed onset of egg production or even complete cessation. Additionally, differential expression of CCDC148 in plasma samples from ovarian cancer patients across various clinical populations was observed by Dufresne et al. (2018). Furthermore, within a significant interval on chromosome 7, we identified another intriguing gene, *NR4A2*, which encodes a member of the steroid-thyroid hormone-retinoid receptor superfamily. Imura et al. (2019) employed genetic fate mapping in mice and demonstrated that early-born and late-born granule cells are localized in the outer and inner sides of the granule cell layer, respectively. They further identified the nuclear orphan receptor *NR4A2* to be preferentially expressed by early-born granule cells. The findings from our study were directly relevant to the traits under investigation.

In this study, we identified a selective interval on chromosome 19 that encompasses two genes, *MSI2* and *NF1*. Previous research by Jessie et al. revealed differential expression of *MSI2* in developing ovaries and oocytes in mice, highlighting the essential roles of *MSI2* and *NF1* genes in healthy follicle and oocyte development and fertility (Sutherland et al., 2015). Additionally, Schafer et al. (1993) demonstrated the important role of neurofibromin, encoded by the *NF1* gene, in the development of the nervous system. Another intriguing gene is *IGF2BP1*, which has previously been shown to influence breast muscle weight and growth traits in chickens (Tan et al., 2021; Wang et al., 2022; Tan et al., 2023; Tan et al., 2024). Drouilhet et al. (2013) found a strong linkage between the multiovulation major gene *FecL*, which affects ovulation rate, and *IGF2BP1*. Moreover, the known target genes of *IGF2BP1* play crucial roles in follicular function, indirectly affecting the development and maturation of ovarian follicles.

Considering the connection between ovarian follicle development and AFE in chickens, abnormalities or disruptions in ovarian and follicle development can cause delays or instability in the reproductive cycle, leading to a delay in egg laying. Therefore, the aforementioned genes, which play important roles in ovarian follicle development, could be associated with genes influencing AFE in broiler breeders. Furthermore, hormones play a crucial role in regulating the reproductive cycle and AFE in chickens. However, unfortunately, we did not locate genes related to the regulation of GnRH, follicle-stimulating hormone (**FSH**), or other hormones.

## CONCLUSIONS

In conclusion, our study revealed a strong genetic correlation between two-stage egg weight and the genetic relationships among AFE, EW, and EN. Based on GWAS analysis, meta-analysis, and selective scanning analysis of 6,842 individuals from both paternal and maternal lines, we identified two notable regions on GGA4 that exhibit significant associations with EW. Additionally, multiple SNPs and genes are likely to be significantly associated with AFE. These findings contribute to a better understanding of the genetic basis of egg-related traits and provide insights into future directions for breeding chickens with the desired EW.

## Appendix Supplementary materials

Image, application 1

## Footnotes

Supplementary material associated with this article can be found in the online version at doi:10.1016/j.psj.2024.103613.
