# Supplementary material for: New insights into the genetic loci related to egg weight and age at first egg traits in broiler breeder
Source: Poult Sci. 2024 Mar 5;103(5):103613. doi: 10.1016/j.psj.2024.103613 (PMC10959720; doi:10.1016/j.psj.2024.103613)

**Table S1** Annotation of significant SNPs associated with the paternal and maternal lines.

| **Lines** | **traits** | **GGA^a^** | **Position ^b^** | **Alleles** | **b^C^** | ***P* value** | **Gene** | **Gap** |
| --- | --- | --- | --- | --- | --- | --- | --- | --- |
| Maternal | AFE | 7 | 21676666 | C/G | -1.46 | 1.70E-06 | *ENSGALG00010021161* | Synonymous variant |
|  |  | 19 | 6694125 | A/G | -0.96 | 1.77E-06 | *SLC6A4* | Intron variant |
|  |  | 27 | 3244969 | A/G | -1.09 | 1.69E-06 | *PHOSPHO1* | 3_prime_UTR |
|  |  | 27 | 3244969 | A/G | -1.09 | 1.69E-06 | *ABI3* | Intron variant |
|  |  | 27 | 3265541 | C/T | -1.02 | 1.80E-06 | *IGF2BP1* | Intron variant |
|  |  | 27 | 3289538 | A/G | -0.96 | 1.52E-06 | *GIP* | Intron variant |
|  | EW32W | 4 | 74693221 | G/C | -0.64 | 8.58E-07 | *SLIT2* | Intron variant |
|  |  | 4 | 75428242 | T/C | -0.68 | 1.31E-07 | *NCAPG* | Intron variant |
|  |  | 4 | 75414844 | A/G | -0.63 | 8.67E-07 | *LCORL* | 3_prime_UTR |
|  | EW43W | 1 | 193234052 | A/G | -1.25 | 6.55E-07 | *ACER3* | Intron variant |
|  |  | 1 | 35380569 | A/C | -0.69 | 1.82E-06 | *NUP107* | Intron variant |
|  |  | 2 | 21866551 | A/G | 0.97 | 5.10E-07 | *CDK14* | Intron variant |
|  |  | 2 | 21568642 | C/T | 0.90 | 1.34E-07 | *STEAP2* | Intron variant |
|  |  | 4 | 471007 | C/T | -0.60 | 1.84E-06 | *OPHN1* | Intron variant |
|  |  | 4 | 547641 | G/C | -0.59 | 1.7E-06 | *STARD8* | Intron variant |
|  |  | 5 | 28167137 | A/G | -0.68 | 8.10E-07 | *RAD51B* | Intron variant |
|  |  | 8 | 23625177 | G/A | -0.70 | 1.51E-06 | *FAF1* | Intron variant |
|  |  | 11 | 5901419 | C/A | -0.61 | 8.03E-07 | *NKD1* | Intron variant |
|  |  | 15 | 1916165 | G/A | -0.73 | 1.22E-06 | *FBRSL1* | Intron variant |
|  |  | 24 | 3217862 | A/G | -0.62 | 1.21E-06 | *UBASH3B* | Intron variant |
| Paternal | EW32w | 4 | 84797258 | C/T | 1.59 | 1.71E-06 | *ENSGALG00010018679* | Intron variant |
|  |  | 7 | 27748531 | C/T | -1.11 | 1.67E-06 | *ENSGALG00010020453* | Intron variant |
|  | EW43W | 4 | 226725 | C/T | -1.08 | 1.63E-06 | *VSIG4* | Intron variant |
|  |  | 4 | 319175 | C/T | -1.08 | 1.63E-06 | *ENSGALG00010019366* | Exon variant |
|  |  | 4 | 328393 | A/C | -1.08 | 1.63E-06 | *ENSGALG00010019368* | Intron variant |
|  |  | 4 | 340494 | T/C | -1.08 | 1.63E-06 | *EDA2R* | Missense variant |
|  |  | 4 | 454225 | A/G | -1.07 | 6.33E-07 | *AR* | 3_prime_UTR |
|  |  | 4 | 460431 | T/C | -1.10 | 5.04E-07 | *OPHN1* | Missense variant |
|  |  | 4 | 460459 | T/G | -1.12 | 5.04E-07 | *OPHN1* | Synonymous variant |
|  |  | 4 | 508586 | T/C | -1.12 | 5.02E-07 | *YIPF6* | 3_prime_UTR |
|  |  | 4 | 531978 | T/C | -1.03 | 2.55E-07 | *STARD8* | Missense variant |
|  |  | 4 | 567765 | G/A | -1.04 | 2.15E-07 | *RXFP2* | Intron variant |
|  |  | 4 | 981776 | A/G | -1.15 | 1.78E-06 | *EFNB1* | 3_prime_UTR |
|  |  | 5 | 12523906 | G/A | -1.00 | 2.01E-06 | *OSBPL5* | Intron variant |
|  |  | 10 | 19125256 | G/C | 1.02 | 1.95E-06 | *PIAS1* | Intron variant |
|  |  | 19 | 7348326 | G/T | 0.89 | 1.39E-06 | *SLC47A1* | Intron variant |

^a^ Gallus gallus chromosome

^b^ Gallus_gallus-7b source

^c^ Allele substitution effect was the additive effect estimated by GCTA

**Table S2** The SNPs identified in the meta-analysis for AFE and EW.

| traits | GGA^a^ | Position^b^ | Alleles | b^c^ | *P* meta | Gene | Gap |
| --- | --- | --- | --- | --- | --- | --- | --- |
| AFE | 7 | 35644583 | T/C | -5.06 | 4.19E-07 | *NR4A2* | 3_prime_UTR |
|  | 7 | 35703733 | A/G | 5.40 | 6.66E-08 | *GPD2* | Synonymous variant |
|  | 7 | 35846854 | A/G | 4.91 | 8.93E-07 | *ACVR1C* | 3_prime_UTR |
|  | 7 | 35905782 | C/G | 5.56 | 2.68E-08 | *ACVR1* | Intron variant |
|  | 7 | 35926036 | T/C | -4.76 | 1.99E-06 | *CCDC148* | 3_prime_UTR |
|  | 7 | 36078197 | C/G | 4.88 | 1.08E-06 | *TANC1* | 5_prime_UTR |
|  | 7 | 36143258 | T/C | -5.08 | 3.85E-07 | *WDSUB1* | Intron variant |
|  | 7 | 36259232 | T/C | 5.05 | 4.46E-07 | *BAZ2B* | Intron variant |
|  | 19 | 9415681 | T/C | -4.95 | 7.53E-07 | *MSI2* | Intron variant |
|  | 19 | 9548348 | T/C | -5.23 | 1.69E-07 | *NF1* | Intron variant |
|  | 19 | 9585655 | A/G | 4.89 | 1.04E-06 | *WSB1* | Intron variant |
|  | 19 | 9619508 | A/T | -4.85 | 1.25E-06 | *KSR1* | Intron variant |
|  | 19 | 9690449 | C/G | 4.81 | 1.49E-06 | *NOS2* | Missense variant |
|  | 19 | 9744092 | T/C | 4.85 | 1.26E-06 | *LYRM9* | Missense variant |
|  | 19 | 9770949 | A/G | -4.76 | 1.93E-06 | *NLK* | Intron variant |
|  | 27 | 3267827 | T/C | -4.99 | 5.97E-07 | *IGF2BP1* | Intron variant |
|  | 27 | 3296091 | A/G | -4.80 | 1.63E-06 | *GIP* | Intron variant |
| 32W | 1 | 34170560 | T/C | 4.81 | 1.53E-06 | *MSRB3* | Intron variant |
|  | 3 | 106968548 | A/G | 5.21 | 1.88E-07 | *MTMR9* | Intron variant |
|  | 4 | 322967 | A/G | 4.97 | 6.67E-07 | *ENSGALG00010016853* | Intron variant |
|  | 4 | 340742 | A/C | -4.86 | 1.16E-06 | *EDA2R* | Missense variant |
|  | 4 | 366869 | T/C | -5.34 | 9.48E-08 | *ENSGALG00010019604* | Exon variant |
|  | 4 | 449781 | T/C | -5.08 | 3.75E-07 | *AR* | 3_prime_UTR |
|  | 4 | 464864 | A/G | -5.43 | 5.62E-08 | *OPHN1* | Synonymous variant |
|  | 4 | 505720 | T/C | 5.75 | 9.19E-09 | *YIPF6* | Intron variant |
|  | 4 | 514684 | A/C | 5.73 | 9.82E-09 | *STARD8* | 5_prime_UTR |
|  | 4 | 565431 | T/C | -5.47 | 4.56E-08 | *RXFP2* | Synonymous variant |
|  | 4 | 74188816 | A/G | 4.95 | 7.43E-07 | *KCNIP4* | Intron variant |
|  | 4 | 74693221 | C/G | 5.08 | 3.81E-07 | *SLIT2* | Intron variant |
|  | 4 | 75389203 | A/G | 5.60 | 2.02E-09 | *LCORL* | Intron variant |
|  | 4 | 75430610 | A/T | -5.60 | 6.78E-09 | *NCAPG* | Intron variant |
|  | 23 | 5535952 | A/G | -4.80 | 1.41E-06 | *TXLNA* | Intron variant |
|  | 23 | 5539456 | A/G | 4.80 | 1.41E-06 | *KPNA6* | 3_prime_UTR |
| 43W | 1 | 35380773 | T/C | 4.77 | 1.82E-06 | *NUP107* | Intron variant |
|  | 1 | 35576092 | A/G | 5.47 | 4.40E-08 | *FRS2* | Intron variant |
|  | 1 | 35672129 | C/G | 4.88 | 1.05E-06 | *BEST3* | Missense variant |
|  | 1 | 35691833 | T/C | -4.80 | 1.55E-06 | *RAB3IP* | Intron variant |
|  | 2 | 21284553 | A/G | 5.54 | 3.02E-08 | *ZNF804B* | Missense variant |
| 43W | 2 | 21566816 | A/T | -5.53 | 3.21E-08 | *STEAP2* | Intron variant |
|  | 2 | 21588925 | T/G | -5.42 | 5.92E-08 | *CFAP69* | Intron variant |
|  | 2 | 21623215 | A/T | 5.46 | 4.85E-08 | *GTPBP10* | Synonymous variant |
|  | 2 | 21640217 | A/G | 5.55 | 2.81E-08 | *CLDN12* | 3_prime_UTR |
|  | 2 | 21756526 | A/T | -5.40 | 6.81E-08 | *CDK14* | Intron variant |
|  | 2 | 22041052 | T/C | 4.77 | 1.86E-06 | *FZD1* | 3_prime_UTR |
|  | 4 | 226725 | T/C | 4.86 | 1.16E-06 | *VSIG4* | Intron variant |
|  | 4 | 296228 | T/C | 4.83 | 1.37E-06 | *GPR83L* | Intron variant |
|  | 4 | 324847 | T/C | -6.21 | 5.19E-10 | *ENSGALG00010016853* | Intron variant |
|  | 4 | 340494 | T/C | -6.19 | 5.87E-10 | *EDA2R* | Missense variant |
|  | 4 | 399801 | A/G | 6.59 | 4.56E-11 | *ENSGALG00010019604* | Intron variant |
|  | 4 | 428848 | A/G | -5.73 | 1.01E-08 | *AR* | Synonymous variant |
|  | 4 | 460431 | T/C | -6.03 | 1.63E-09 | *OPHN1* | Missense variant |
|  | 4 | 507737 | T/C | 6.46 | 1.08E-10 | *YIPF6* | 3_prime_UTR |
|  | 4 | 522234 | T/C | -6.75 | 1.5E-11 | *STARD8* | Intron variant |
|  | 4 | 565387 | T/C | -5.67 | 1.46E-08 | *RXFP2* | Missense variant |
|  | 4 | 75404361 | A/T | -5.80 | 6.69E-09 | *LCORL* | Intron variant |
|  | 4 | 75427309 | T/G | 4.79 | 1.68E-06 | *NCAPG* | Intron variant |
|  | 5 | 28167155 | T/C | 6.09 | 1.11E-09 | *RAD51B* | Intron variant |
|  | 5 | 28447168 | A/G | 4.95 | 7.58E-07 | *ZFYVE26* | Intron variant |
|  | 8 | 23625177 | A/G | 4.81 | 1.51E-06 | *FAF1* | Intron variant |
|  | 11 | 5901419 | A/C | 5.57 | 2.50E-08 | *NKD1* | Intron variant |
|  | 19 | 7348320 | T/C | -4.76 | 1.93E-06 | *SLC47A1* | Intron variant |
|  | 24 | 3217664 | A/G | 4.90 | 9.48E-07 | *UBASH3B* | Intron variant |

^a^ Gallus gallus chromosome

^b^ Gallus_gallus-7b source

^c^ Allele substitution effect was the additive effect estimated by Me

**Table S3** The effect of different blocks on the EW phenotype of broiler breeders.

| Block | snp^a^ | Tag SNPs^b^ | Genotype | N^c^ | EW32W _EBV^d^ | *P* value^e^ | EW43W _EBV^d^ | *P* value^e^ |
| --- | --- | --- | --- | --- | --- | --- | --- | --- |
| Block1 | 14 | 4_462248 | GG | 310 | -0.410 | 2e-05 | -0.418 | 4.5e-04 |
|  |  |  | TG | 1145 | -0.122 |  | -0.132 |  |
|  |  |  | TT | 1265 | 0.2108 |  | 0.222 |  |
| Block2 | 20 | 4_497796 | AA | 281 | -0.416 | 3.2e-05 | -0.446 | 5.8e-04 |
|  |  |  | GA | 1137 | -0.131 |  | -0.129 |  |
|  |  |  | GG | 1302 | 0.2044 |  | 0.209 |  |
| Block3 | 6 | 4_507737 | CC | 274 | -0.381 | 7.3e-05 | -0.459 | 5.9e-04 |
|  |  |  | TC | 1143 | -0.139 |  | -0.124 |  |
|  |  |  | TT | 1303 | 0.202 |  | 0.205 |  |
| Block4 | 14 | 4_517679 | CC | 1603 | 0.1249 | 1.5e-03 | 0.140 | 3.4e-03 |
|  |  |  | CT | 948 | -0.144 |  | -0.154 |  |
|  |  |  | TT | 169 | -0.379 |  | -0.462 |  |
| Block5 | 2 | 4_517957 | AA | 136 | -0.167 | 0.14 | -0.323 | 0.073 |
|  |  |  | GA | 927 | -0.105 |  | -0.129 |  |
|  |  |  | GG | 1657 | 0.072 |  | 0.098 |  |
| Block6 | 3 | 4_526796 | AA | 170 | -0.372 | 1.4e-03 | -0.450 | 3.6e-03 |
|  |  |  | TA | 948 | -0.147 |  | -0.157 |  |
|  |  |  | TT | 1602 | 0.1264 |  | 0.141 |  |
| Block7 | 33 | 4_565431 | CC | 1492 | 0.182 | 3e-05 | 0.205 | 1.3e-04 |
|  |  |  | CT | 1022 | -0.180 |  | -0.205 |  |
|  |  |  | TT | 206 | -0.422 |  | -0.473 |  |
| Block8 | 469 | 4_75351462 | AA | 1120 | 0.2350 | 2.3e-06 | 0.229 | 6.7e-05 |
|  |  |  | AG | 1239 | -0.110 |  | -0.079 |  |
|  |  |  | GG | 361 | -0.354 |  | -0.439 |  |

^a^ Number of SNPs in each block.

^b^ SNP with the highest p-value in each block

^c^ Number of individuals per genotype

^d^ Breeding values calculated using GBLUP

^e^ P-value for each block individually tested for significance

**Table S4** The effect of different blocks on the AFE phenotype of broiler breeders.

| Block | SNP^a^ | TagSNP^b^ | Genotype | N^c^ | AFE | AFE EBV^d^ | Pvalue^e^ |
| --- | --- | --- | --- | --- | --- | --- | --- |
| Block 1 | 158 | 7_35660624 | AA | 216 | 181.76 | -0.5823 | 2.7e-04 |
|  |  |  | GA | 1013 | 182.03 | -0.2236 |  |
|  |  |  | GG | 1483 | 182.98 | 0.23752 |  |
| Block 2 | 69 | 7_35750007 | AA | 284 | 181.58 | -0.5591 | 1.5e-04 |
|  |  |  | GA | 1144 | 182.15 | -0.1881 |  |
|  |  |  | GG | 1284 | 183.08 | 0.29123 |  |
| Block 3 | 152 | 7_35772256 | CC | 214 | 181.79 | -0.5608 | 1.7e-04 |
|  |  |  | GC | 1009 | 182.00 | -0.2444 |  |
|  |  |  | GG | 1489 | 182.98 | 0.24620 |  |
| Block 4 | 18 | 7_35801064 | CC | 278 | 181.66 | -0.5151 | 9.3e−05 |
|  |  |  | TC | 1139 | 182.10 | -0.2204 |  |
|  |  |  | TT | 1295 | 183.09 | 0.30451 |  |
| Block 5 | 3 | 7_35846742 | CC | 148 | 182.86 | -0.0409 | 1.6e−08 |
|  |  |  | TC | 841 | 181.35 | -0.6275 |  |
|  |  |  | TT | 1723 | 183.07 | 0.30979 |  |
| Block 6 | 9 | 7_35849057 | AA | 226 | 181.70 | -0.5364 | 7.9e−05 |
|  |  |  | GA | 1045 | 182.06 | -0.2480 |  |
|  |  |  | GG | 1441 | 182.99 | 0.2639 |  |
| Block 7 | 33 | 7_35905782 | CC | 1430 | 183.00 | 0.2792 | 2.9e−05 |
|  |  |  | CG | 1053 | 182.06 | -0.2477 |  |
|  |  |  | GG | 229 | 181.65 | -0.6046 |  |
| Block 8 | 9 | 7_36078197 | CC | 1430 | 183.01 | 0.2792 | 2.9e−05 |
|  |  |  | CG | 1053 | 182.06 | -0.2477 |  |
|  |  |  | GG | 229 | 181.65 | -0.6046 |  |

Same notes as Table S3

**Supplementary Files**

**Table S1** Annotation of significant SNPs associated with the paternal and maternal lines.

**Table S2** The SNPs identified in the meta-analysis for AFE and EW.

**Table S3** The effect of different blocks on the EW phenotype of broiler breeders.

**Table S4** The effect of different blocks on the AFE phenotype of broiler breeders.

**Figure S1** The SNP density maps before and after gene imputation. (A) Plot of the density distribution of SNPs on chromosomes before filling. (B) Density distribution of SNPs on chromosomes after filling using resequencing.

**Figure S2** The line chart of cross-validation errors. For the coefficient of variation value for each K-value, the accessions were divided into four subgroups.

**Figure S3** The LD decay in paternal and maternal lines.

**Figure S4** The Manhattan and quantile–quantile (Q‒Q) plots of the GWAS for AFE and EW of paternal and maternal lines. The Manhattan diagrams in A, B, and C represent the AFE, EW32W, and EW43W traits of the maternal line. D, E, F represent the AFE, EW32W, and EW43W traits of the paternal line. In the Manhattan plots, each dot represents an SNP. The blue and red horizontal lines represent genome-wide significance thresholds and potential suggestive thresholds. G, H, and I represent Q-Q plots of the corresponding trait in the maternal line, and J, K, and L represent Q-Q plots of the corresponding trait in the paternal line. The red line represents the concordance of observed and expected values. The genomic inflation factor (λ). These results demonstrate the high accuracy and reliability of the GWAS results.

**Figure S5** Results of selection signal analysis of EW in the candidate interval of chromosome 4. Results of *F*_ST_ and π ratio analyses of paternal and maternal lines.

**Figure S6** Results of selection signal analysis of AFE in the candidate interval of chromosome 19. Results of *F*_ST_ and π ratio analyses of paternal and maternal lines.


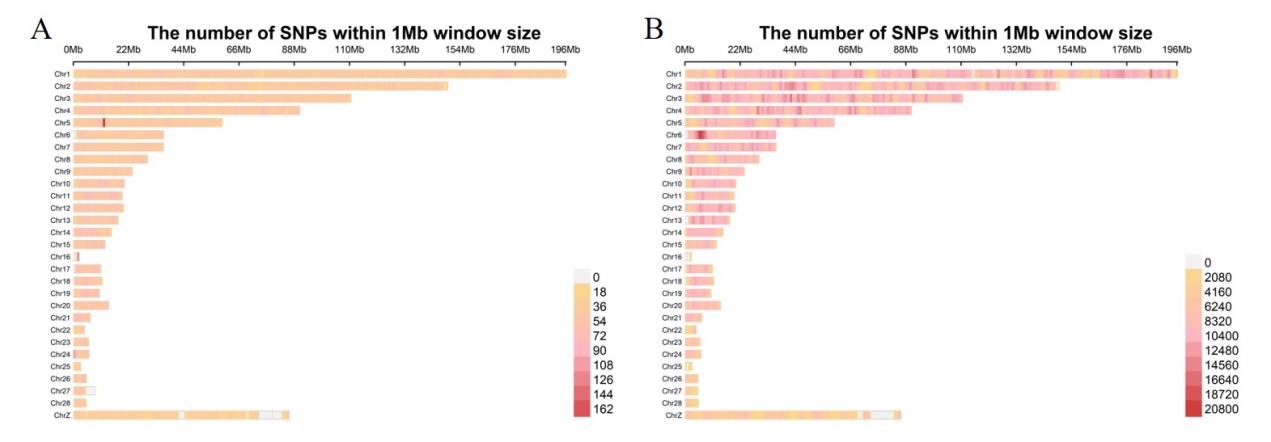


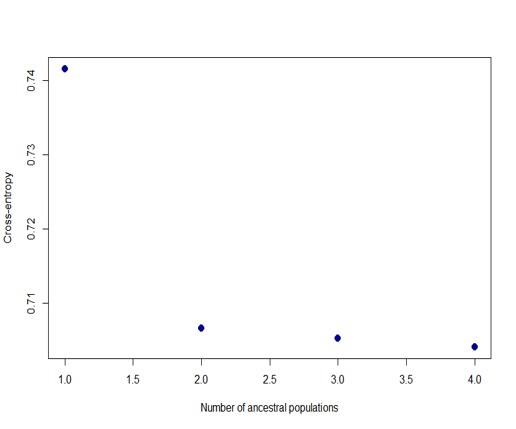


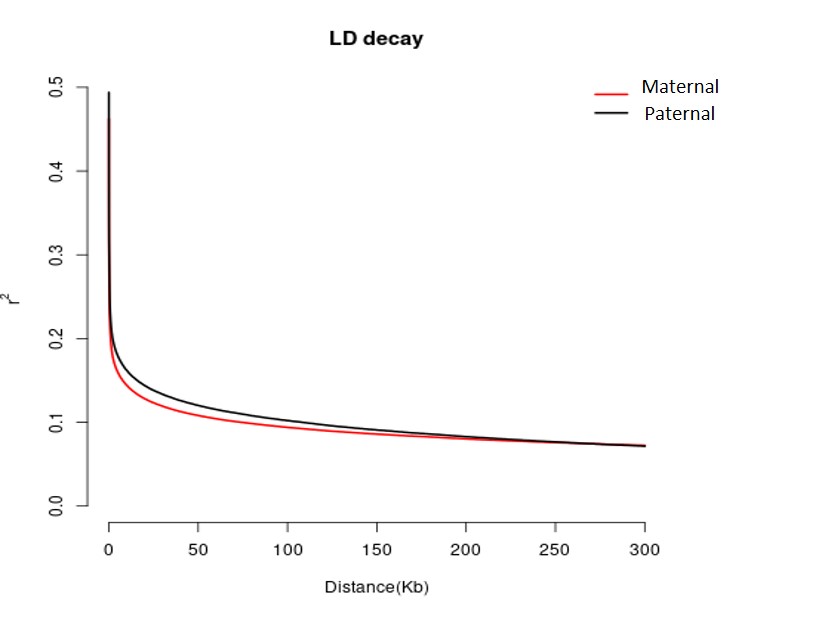


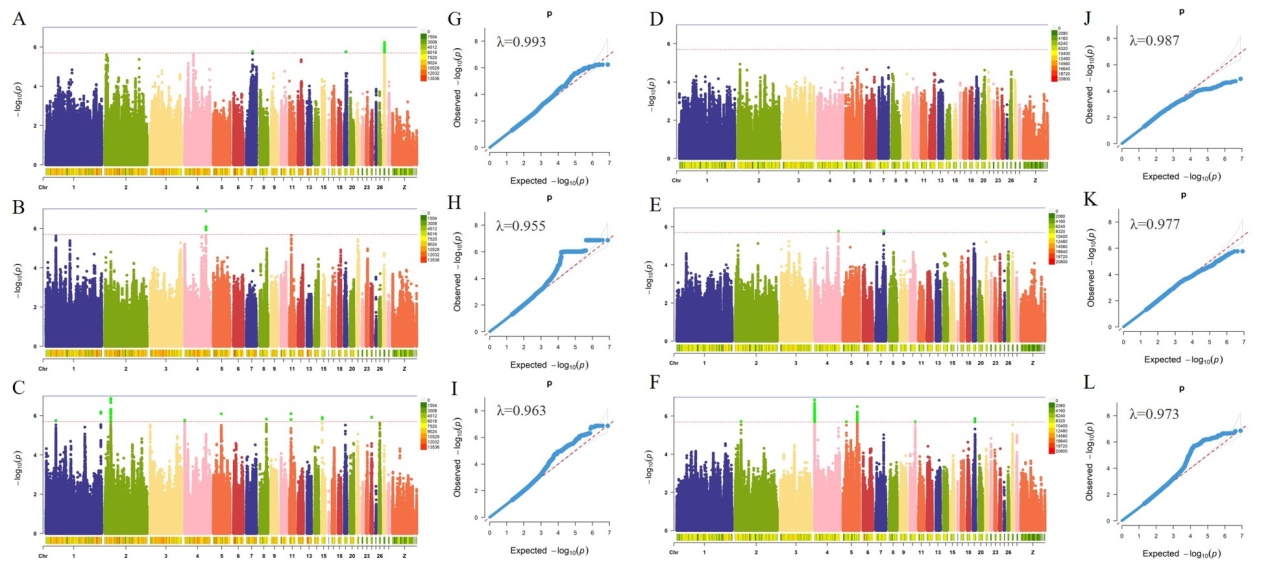


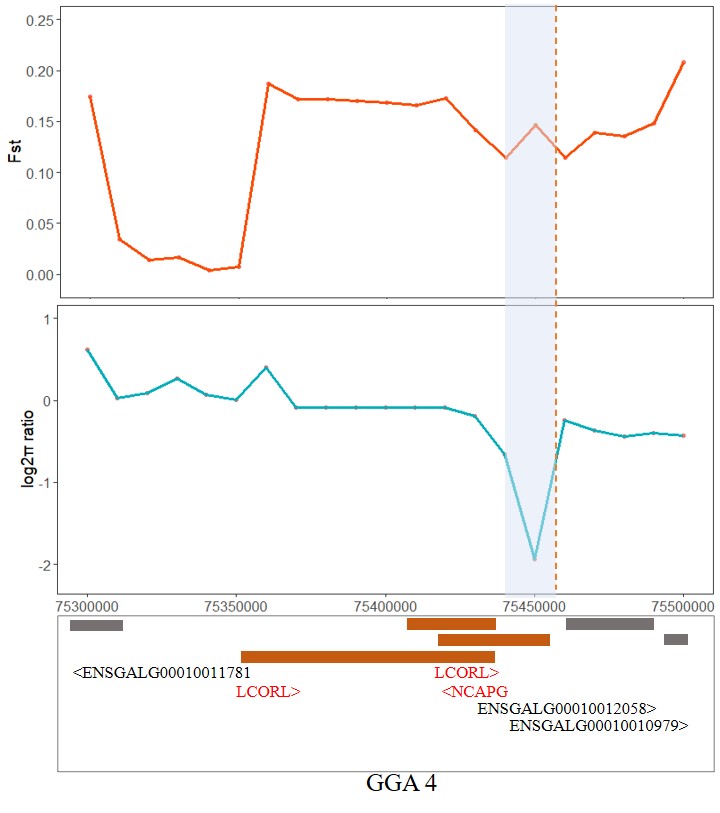


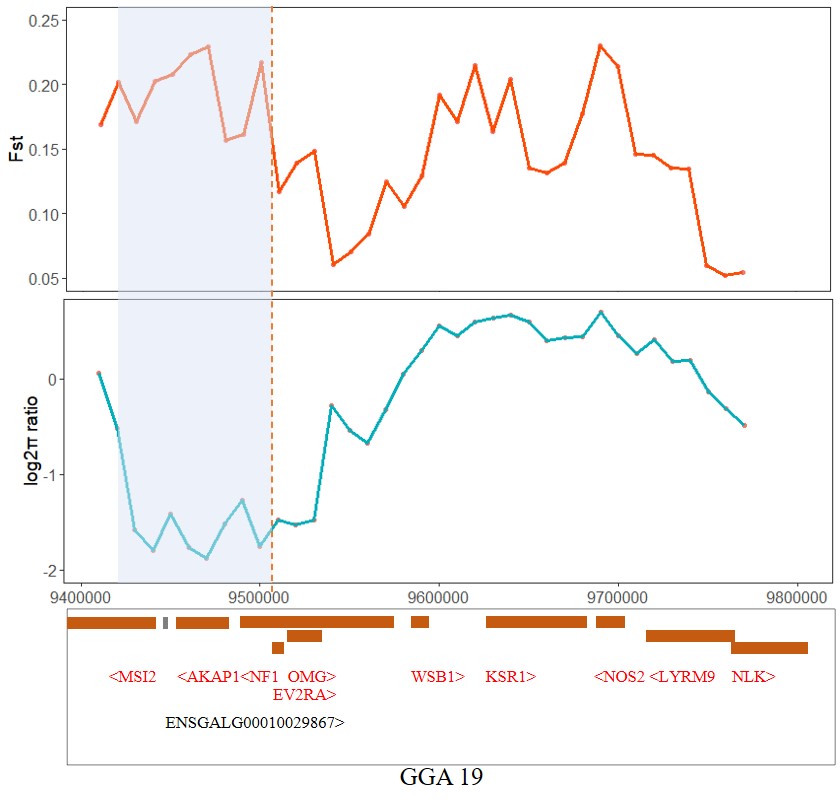

Supplement: Supplementary file 1 [file mmc1.docx]
